# Significance of Malic Enzyme 1 in Cancer: A Review

**DOI:** 10.3390/cimb47020083

**Published:** 2025-01-29

**Authors:** Rina Fujiwara-Tani, Chie Nakashima, Hitoshi Ohmori, Kiyomu Fujii, Yi Luo, Takamitsu Sasaki, Ruiko Ogata, Hiroki Kuniyasu

**Affiliations:** Department of Molecular Pathology, Nara Medical University School of Medicine, 840 Shijo-cho, Kashihara 634-8521, Japan; c-nakashima@naramed-u.ac.jp (C.N.); brahmus73@hotmail.com (H.O.); toto1999-dreamtheater2006-sms@nifty.com (K.F.); lynantong@hotmail.com (Y.L.); takamitu@fc4.so-net.ne.jp (T.S.); pkuma.og824@gmail.com (R.O.)

**Keywords:** malic enzyme 1, cancer stem cells, energy metabolism, epithelial–mesenchymal transition, hypoxia

## Abstract

Malic enzyme 1 (ME1) plays a key role in promoting malignant phenotypes in various types of cancer. ME1 promotes epithelial–mesenchymal transition (EMT) and enhances stemness via glutaminolysis, energy metabolism reprogramming from oxidative phosphorylation to glycolysis. As a result, ME1 promotes the malignant phenotypes of cancer cells and poor patient prognosis. In particular, ME1 expression is promoted in hypoxic environments associated with hypoxia-inducible factor (HIF1) α. ME1 is overexpressed in budding cells at the cancer invasive front, promoting cancer invasion and metastasis. ME1 also generates nicotinamide adenine dinucleotide (NADPH), which, together with glucose-6-phosphate dehydrogenase (G6PD) and isocitrate dehydrogenase (IDH1), expands the NADPH pool, maintaining the redox balance in cancer cells, suppressing cell death by neutralizing mitochondrial reactive oxygen species (ROS), and promoting stemness. This review summarizes the latest research insights into the mechanisms by which ME1 contributes to cancer progression. Because ME1 is involved in various aspects of cancer and promotes many of its malignant phenotypes, it is expected that ME1 will become a novel drug target in the near future.

## 1. Introduction

ME1, also known as cytosolic NADP+-dependent malic enzyme and malate dehydrogenase, is a multifunctional protein that links glycolysis and the tricarboxylic acid cycle (TCA cycle) by directly converting the malate produced in the TCA cycle to pyruvate [1]. ME1 is involved in glycolysis, the TCA cycle, nicotinamide adenine dinucleotide phosphate (NADPH) production, glutamine metabolism, and lipogenesis, specifically through the decarboxylation of malate to form pyruvate and ultimately NADPH [2].

NADPH produced by ME1 plays important roles in other biological processes, including antioxidant stress and detoxification pathways, as well as the generation of reactive oxygen species (ROS). ME1, isocitrate dehydrogenase 1 (IDH1), and two enzymes of the pentose phosphate pathway (PPP), glucose-6-phosphate dehydrogenase (G6PD) and 6-phosphogluconate dehydrogenase (6PGD), are involved in maintaining an adequate pool of NADPH [3,4,5].

ME1 was among the first insulin-regulated genes to be identified in liver and adipose tissue and is transcriptionally regulated by thyroxine [6,7]. ME1 activity is reciprocally and dynamically regulated by post-translational modifications such as K^337^ acetylation, which enhances its activity, and S^336^ phosphorylation, which inhibits it. These modifications affect the NADPH/NADP ratio and fatty acid synthesis [8].

Although ME1 is overexpressed in various cancers, its role in cancer tumorigenesis and progression remains to be fully elucidated.

## 2. Discovery of ME1

In the 1950s, it was thought that in addition to the pentose phosphate pathway (PPP), another enzyme that decarboxylates malate to produce NADPH was required to supply NADPH to the high fatty acid synthesis activity observed in the liver and adipose tissue. Ochoa et al. identified an activity that decarboxylates malate to produce pyruvate in an NADP⁺-dependent manner, and named it NADP⁺-malic enzyme (ME1) [9]. Subsequently, ME2 and ME3, which exist in mitochondria and are dependent on NAD+ and NADP+, respectively, were identified [10]. ME1 is mainly present in the cytoplasm and catalyzes the following reaction [11].

## 3. Regulation of ME1 Expression

The regulation of ME1 expression in cancer is linked to p53 and KRAS mutations. Wild-type p53 suppresses ME1 expression, making ME1 overexpression common in cancers with p53 mutations [12]. Human spot 14 protein modulates p53 target genes through direct interactions with the thyroid receptor or other p53 coactivators such as Zac1, and also regulates ME1 promoter activity [13].

KRAS mutations also upregulate ME1 expression in hepatocellular carcinoma (HCC) [14], non-small-cell lung cancer (NSCLC) [15], and colorectal cancer (CRC) [16].

As an oxidative stress-related gene, ME1 is also regulated by the Kelch-like ECH-associated protein 1 (Keap1), the nuclear factor erythroid 2-related factor 2 (NRF2) oxidative stress response pathway [17], and BTB and CNC homology 1 (Bach-1), a mediator of oxidative stress responses [18].

Other regulators of ME1 expression include the canonical Wnt signaling pathway in breast cancer [19], dietary factors such as a high-fat diet in intestinal and hepatic tissues [20], and signaling through peroxisome proliferator-activated receptor (PPAR)/early growth response protein 4 (EGR4) [21].

Epigenetic regulation also plays a role in controlling ME1 expression. For instance, RNA m5C methyltransferase NSUN2 mediates 5-methyl cytosine (m5C) methylation, stabilizing ME1 and promoting metabolic reprogramming and cell cycle progression [22]. Deacetylase sirtuin 2 (SIRT2) mediates the deacetylation of phosphoglycerate mutase 5 (PGAM5), activating ME1, leading to ME1 dephosphorylation, which subsequently drives lipid accumulation and proliferation in HCC cells [23].

MicroRNAs (miRNAs) further contribute to ME1 regulation. Examples include miR-30a [16], miR-30c-5p [24], miR-612 [25], and miR-885-5p [26], which target ME1 and influence its expression in various cancer contexts.

## 4. ME1 and Malignancy

ME1 is overexpressed in many cancers, contributing to poorer prognosis and more aggressive tumor behavior. In HCC, ME1 is highly expressed in intermediate cell subtypes [27], and its overexpression is associated with lower overall and progression-free survival rates compared with HCC with normal ME1 levels [14]. ME1 is also a poor prognostic factor for lipid metabolism in HCC [28,29], in which its overexpression can lead to epithelial–mesenchymal transition (EMT) [14] and anticancer drug resistance [30] in HCC. Finally, ME1 is key in the transformation from nonalcoholic steatohepatitis (NASH) to HCC [31].

In gastric cancer, ME1 promotes cell proliferation and metastasis [32], and its overexpression in CRC is associated with cancer progression [33], while it promotes colon carcinogenesis in Apc^Min/+^ mice [34].

In breast cancer, ME1 overexpression correlates with proliferation, lymph node metastasis, vascular invasion, and poor prognosis. This is because of enhanced survival rate, motility, EMT, and reduced ROS levels [35]. ME1 is particularly associated with the malignant phenotypes of basal-like breast cancer [36]. ME1 expression is also significantly correlated with speedy/RINGO cell cycle regulator family member C (SPDYC), which influences the tumor immune microenvironment and lipid metabolism [37].

In NSCLC, ME1 is more strongly expressed in squamous cell carcinoma (SCC) than in adenocarcinoma and is correlated with smoking and poor prognosis [15,38].

In oral SCC (OSCC), ME1 is overexpressed in 48% of cases and correlates with tumor factors such as T stage, N stage, clinical stage, and histological grade. ME1 overexpression is also associated with poor prognosis [39] and increased malignancy, particularly invasiveness driven by EMT at the invasive front [39,40]. ME1 promotes cancer progression by altering metabolism and stemness, which then increases tumor growth and invasion [39,40].

ME1 is overexpressed in laryngeal SCC [41] and promotes the motility and invasiveness of nasopharyngeal carcinoma cells [42]. In non-solid tumors, ME1 overexpression leads to reduced ROS levels and drug resistance [43], which contribute to poor prognosis.

In acute myelocytic leukemia, melanoma, and papillary renal cell carcinoma, ME1 overexpression is similarly correlated with poor prognosis [44,45].

## 5. ME1 and Benign Diseases

ME1 is involved in several benign diseases, including metabolic syndrome and diabetes [46]. ME1 may induce hyperinsulinemia, hyperglycemia, inflammation, and oxidative stress in patients with obesity and type 2 diabetes and is a risk factor for the development of malignant tumors in these diseases [46,47]. Abnormal tubular lipid metabolism in diabetes is a risk factor for diabetic nephropathy, and ME1 is a risk factor for altered lipid metabolism [48,49]. ME1 is involved in the PPAR signaling pathway and is associated with hypertension risk [50]. ME1 also promotes cellular senescence in inflammatory diseases [51]. ME1 is also hypomethylated in endometriosis, making it a useful disease marker [49].

## 6. ME1 and Malignant Phenotypes

ME1 is involved in various malignant phenotypes. It enhances proliferation, cell motility, invasion, colony formation in soft agar, glucose consumption, lactate production, NADPH, and EMT [12,14,15,16,25,32,34,36,42,43,44,45,46,47,48,49,50,51,52,53,54,55,56]. Conversely, ME1 reduces senescence, ROS, and apoptosis [12,14,32,42,52,56]. ME1 also promotes cancer cell proliferation under hypoxic conditions [36]. Animal studies have demonstrated tumor growth in mouse models [16] and increased adenocarcinomas and adenomas in APC^MIN/+^- and ME1-overexpressing mice [8,34].

In gastrointestinal cancer, the ME1 phenotype is characterized by important adipogenic components and paracrine communication between tumors and adjacent non-tumor tissues. This interaction induces lipid and mucin production, which support tumor growth [34,46]. Rather than acting as an independent cancer promoter, ME1 works in conjunction with lipid metabolism, cell proliferation, cell motility promotion, ROS generation related to EMT, NADP recycling enzymes, and redox networks. ME1 is thought to contribute significantly to ROS buffering via NADPH-dependent recycling in the glutathione and thioredoxin pathways [46].

## 7. EMT

EMT involves dynamic changes in cell organization, transitioning from epithelial to mesenchymal phenotypes, which leads to functional changes in cell migration and invasion [57]. EMT is characterized by decreased expression of epithelial cell–cell adhesion molecules such as E-cadherin (ECD) and claudin 4 and increased expression of mesenchymal intermediate fiber vimentin. EMT is involved in disease progression and is associated with poor prognosis in head-and-neck SCC [58], while ME1-induced EMT is prominent in OSCC [39].

In human OSCC cells, suppression of lactate fermentation by knockdown of ME1 and lactate dehydrogenase A or inhibition of pyruvate dehydrogenase (PDH) kinase reduces lactate secretion, increases extracellular pH, and suppresses the EMT phenotype [40]. In contrast, suppressing oxidative phosphorylation by knocking down PDH increases lactate secretion, decreases extracellular pH, and promotes the EMT phenotype [40]. Alterations in extracellular pH are strongly correlated with EMT [59]. Thus, EMT is linked to energy metabolism through glycolysis.

In HCC, silencing ME1 inhibits cell migration and invasiveness by inducing ECD expression and reducing N-cadherin and vimentin expression via an ROS-dependent pathway [11]. Thus, ME1 induces EMT in part by increasing SNAIL expression and suppressing ECD expression [42].

## 8. Budding

Budding at the invasive front of a tumor is correlated with malignant properties in many cancers. Tumor budding refers to the formation of small, undifferentiated cancer cell clusters consisting of up to five cancer cells or a single cancer cell at the invasion front [60]. This phenomenon is linked to the malignant properties of CRC and is used as a prognostic marker [61,62]. In oral cancer, tumor budding is also correlated with lymph node metastasis and worse disease-free and overall survival, making it an important prognostic marker [63,64,65].

Cancer cells exhibiting budding acquire metastatic potential by adopting EMT phenotypes such as reduced cell adhesion and enhanced stemness [66]. However, the EMT during budding is considered partial and incomplete [67]. The Warburg effect and hypoxic environment play important roles in the acquisition of EMT characteristics in budding cancer cells. Budding is correlated with glycolysis promotion via increased expression of glucose transporter type 1 (GLUT1), which promotes glucose uptake [68]. Fructose-1,6-bisphosphatase, which promotes oxidative phosphorylation (OXPHOS), is suppressed by EMT-induced Snail. Consequently, the energy metabolic pathway is reprogrammed toward glycolysis during the EMT process [69]. Hypoxia in the tumor microenvironment (TME) of advanced cancer promotes tumor budding in CRC [70].

Furthermore, chemical hypoxia induced by CoCl_2_ treatment increases ME1 expression, enhances hypoxia-inducible factor (HIF)-1α expression, and promotes the EMT phenotype in OSCC cells [40]. Hypoxia also activates Yes-associated protein (YAP), which is reversed by ME1 knockdown. These findings suggest that in a hypoxic TME, cancer cells at the invasive front may reprogram their energy metabolism via ME1 overexpression, resulting in increased lactate secretion, decreased extracellular pH, and YAP activation. These changes facilitate EMT and subsequent tumor budding [40].

## 9. TME Acidification and Budding

As mentioned above, the Warburg effect, caused by the reprogramming of the energy metabolism of cancer cells, leads to acidification of the TME due to increased lactate secretion. A decrease in extracellular pH strongly correlates with EMT and promotes cancer cell proliferation, migration, invasion, and metastasis in acidic environments through the pH-sensing G protein-coupled receptors (GPCRs) GPR4, GPR65 (T-cell death-associated gene 8 protein, TDAG8), GPR68 (ovarian cancer G-protein coupled receptor 1, OGR1), and GPR132 (G2 accumulation protein, G2A) [59]. Low pH in the cancer TME promotes the infiltration of myeloid-derived suppressor cells, regulatory T cells, and tumor-associated macrophages; induces programmed death ligand 1 (PD-L1) expression in cancer cells; and inhibits T-cell antitumor immunity [59,71]. Additionally, it enhances cancer cell resistance to anticancer drugs [59].

In OSCC, ME1 expression leads to a shift in energy metabolism from OXPHOS to glycolysis, resulting in decreased oxygen consumption and increased lactate production, which in turn promote tumor formation and growth [36,39]. Furthermore, knockdown of monocarboxylate transporter 1 (MCT1), a transporter responsible for lactate secretion, increases extracellular pH [40]. These changes in energy metabolism are closely related to extracellular pH. Along with ME1-mediated reprogramming of energy metabolism [40], extracellular carbonic anhydrase and monocarboxylate transporters play key roles in decreasing the extracellular pH under hypoxic conditions, further promoting cancer cell invasion, metastasis, and stemness [71].

Thus, a hypoxic environment, promotion of the Warburg effect, decreased extracellular pH, and EMT may be involved in tumor budding and the acquisition of a malignant phenotype. Moreover, tumor budding is significantly correlated with ME1 expression, with ME1 levels increasing with cancer progression. Therefore, tumor budding and ME1 expression may be useful markers of OSCC malignancy [40].

## 10. Energy Metabolism in Cancer Cells

In the presence of oxygen, most human cells convert lactate into carbon dioxide and usable energy through OXPHOS, which is localized in the mitochondria [72,73]. In contrast, Otto Warburg discovered the Warburg effect, in which tumors rapidly ferment glucose to lactate, even under oxygen-rich conditions [74,75] This process, unlike OXPHOS, relies on glycolysis, which produces less adenosine triphosphate (ATP) from glucose than OXPHOS [76]. However, glycolysis is more energy-efficient on a per minute basis, producing roughly twice as much energy as OXPHOS despite yielding less ATP per glucose molecule [77].

OXPHOS activity is linked to mitochondrial volume; however, ME1 knockdown increases mitochondrial area while reducing lactate production [39], which in turn promotes the Warburg effect. Cancer cells use ME1-generated pyruvate for lactate fermentation [78,79]. Reducing the glutamine concentration in the culture medium suppresses OSCC cell proliferation, while increasing the glutamine concentration enhances their proliferative ability, even in the absence of glucose. ME1 knockdown prevents glutamine-induced proliferation [39]. This suggests that OSCC cells utilize ME1 to induce glutaminolysis for energy production. A similar dependence on glutamine has also been observed in CRC cells [80].

## 11. Glutamine Metabolism and Redox

In normal human cells, glutamine is converted to α-ketoglutarate, which enters the TCA cycle and is used to generate ATP and produce nucleic acids, lipids, and other amino acids [81,82]. Cancer cells often metabolize glutamine to generate intermediates (e.g., lipids, nucleic acids, and amino acids) needed for the synthesis of these biological components [80].

ME1 knockdown suppresses cell proliferation, particularly under low-glucose and low-glutamine conditions. This effect is accompanied by upregulation of G6PD, a rate-limiting enzyme in the PPP, along with pyruvate kinase (PK) M and acetyl-coenzyme A carboxylase-α (ACC), and a decrease in glutamate dehydrogenase 1 (GLUD1) expression [39,42]. In contrast, ME1 enhances the levels of 6PDG, an enzyme involved in the second PPP reaction [55], thereby promoting tumorigenicity [83]. Thus, ME1 is involved in NADPH production and the conversion of malate to pyruvate, activating both glycolysis and the PPP through a positive-feedback mechanism [39].

The reprogramming of energy metabolism from OXPHOS to aerobic glycolysis suppresses mitochondrial ROS production [84,85]. In OSCC cells, ME1 knockdown increases the NADP/NADPH ratio and decreases the glutathione/glutathione SH (GSH/GSSG) ratio, suggesting that ME1 helps maintain the reducing environment in cancer cells [39]. ME1 depletion also reduces the cells’ tolerance to low-glucose conditions [42] and enhances radiation-induced growth inhibition [56], primarily due to increased ROS levels.

In HCC cells, the transcriptional activation of ME1 by NRF2 helps cells exposed to strong ROS to adapt and survive [86]. In lung SCC, ME1 overexpression strengthens the glycolytic phenotype and increases the dependence on glutaminolysis compared with lung adenocarcinomas [87]. Additionally, the GSH/GSSG ratio is elevated in lung SCC, possibly due to increased NADPH production by ME1 [84].

In gefitinib-resistant NSCLC cell lines, ME1-induced NADPH elevation suppresses apoptosis [88], suggesting that increased NADPH causes drug resistance [86]. ME1 is strongly and consistently associated with NADPH-dependent reductases in NSCLC, highlighting the role of ME1 and G6PD in regulating the redox state and inducing drug resistance by altering NADPH levels [46,89].

NADP/NADPH-dependent reductases, such as prostaglandin reductase 1 (PTGR1) and thioredoxin reductase 1 (TXNRD1), detoxify or activate xenobiotics, metabolize intermediates of endogenous biochemical pathways, regenerate endogenous antioxidants, and mediate the overall redox state of cells. These enzymes contribute to anti-apoptotic survival, EMT, and metastasis, further emphasizing the role of ME1 in promoting cancer progression and resistance.

## 12. ME1 and Stemness

As mentioned earlier, ME1 is strongly associated with EMT, which is accompanied by enhanced stemness in cancer cells [90,91]. Stemness in turn is closely associated with metastatic potential [92,93]. ME1 is involved in several cancers, including gastric cancer [24,30], breast cancer [94], CRC [94], HCC [14,30], bladder cancer [25], and OSCC [39,40]. Therapeutic resistance is a hallmark of cancer stemness [95,96,97]. ME1 overexpression induces radioresistance in head-and-neck SCC and NSCLC [15,56] and has also been reported in acute myelocytic leukemia [43,98], NSCLC [89], HCC [30], and prostate cancer [99]. These findings suggest that ME1 plays a critical role in promoting cancer cell stemness.

The relationship between ME1 and stem cell-related genes is also significant. In APC^MIN/+^ mice, ME1 overexpression enhances colon carcinogenesis, with increased expression of Wnt/Sp5, indicating that ME1 activates the β-catenin/Wnt signaling pathway [34]. In cytogenetically normal acute myeloid leukemia, ME1 activates the interleukin (IL)-6/Janus kinase 2 (JAK2)/signal transducer and activator of transcription 3 (STAT3) pathway, which correlates with poor prognosis [44]. Additionally, ME1-driven glutaminolysis is associated with KRAS mutations [100], which are a metabolic phenotype associated with cancer stem cells (CSCs) and are emerging as a potential therapeutic target [101,102].

ME1 upregulates the activation of YAP and tafazzin (TAZ) through the PPAR signaling pathway in association with glutaminolysis [21]. This activation enhances stemness [100,103]. YAP is activated by ME1-mediated metabolic reprogramming [104]. YAP/TAZ interacts with Snail/Slug to regulate stem cell function and the mesenchymal phenotype expression [105]. YAP is also activated in OSCC, but is abolished by ME1 [40]. YAP activation is a key factor in promoting the EMT phenotype and cancer progression in OSCC [39]. Additionally, G6PD enhances stemness via the Krüppel-like factor 5 (KLF5) pathway [106].

Interestingly, a mutant ME1 protein lacking enzymatic activity still promoted cell proliferation and colony formation in wild-type ME1-silenced cells, suggesting that ME1 may enhance stemness through 6PGD activation [55].

ME1 also contributes to the production of NADPH, which plays a role in maintaining stemness through redox maintenance [107]. ME1 is co-expressed with two other cytoplasmic NADPH-generating enzymes (G6PD and IDH1), along with other cytoplasmic enzymes that utilize NADPH as an electron donor, such as PTGR1 and NAD(P)H quinone dehydrogenase 1 (NQO1), and antioxidant stress-related proteins such as tripartite motif-containing 16 (TRIM16) [47]. These redox-related genes may play a role in promoting stemness. In OSCC, NQO1 expression is regulated by sponging microRNA-494, involving homeobox protein Hox-A11 antisense RNA (HOXA11-AS), a long non-coding RNA (lncRNA) belonging to the homeobox (HOX) gene cluster that promotes liver metastasis in CRC [108,109,110].

## 13. Hypoxia and HIF1α

As noted above, the relationship between ME1 and budding is critical for cancer invasive fronts that exhibit budding. This is significant because cancer cells at these invasive fronts infiltrate stromal tissues with low vascular density, making them susceptible to hypoxic conditions. Hypoxic cancers are more invasive and have a worse prognosis than non-hypoxic cancers [111]. Thus, hypoxia is closely related to the promotion of tumor budding phenotypes. In oral malignant melanoma, hypoxia occurs at the invasion front, accompanied by increased HIF1α expression [112]. Under hypoxic conditions, HIF1α is stabilized and activated, leading to the upregulation of key factors, including vascular endothelial growth factor (VEGF), which drives angiogenesis, matrix metallopeptidase 9 (MMP9), and MMP7, which enhance cancer invasion, and EMT-related genes [100,112,113,114]. Additionally, increased ROS also stabilizes HIF1α [115].

Simultaneous upregulation of HIF1α and ME1 is observed at the cancer front [40], with ME1 expression being induced by hypoxia [36,40]. HIF1α knockdown reduces ME1 expression [116], indicating that ME1 is a target gene of HIF1α and that its overexpression at the invasive front is part of the hypoxic response. Hypoxia also leads to mitochondrial dysfunction, reducing the mitochondrial membrane potential, inhibiting OXPHOS, and lowering oxidative stress [40]. Thus, a hypoxic environment reprograms energy metabolism, suppresses OXPHOS, and promotes glycolysis and lactate fermentation, thereby contributing to altered cancer phenotypes [117]. HIF1α activity further enhances glycolytic flux [118,119,120].

Hypoxia-induced HIF1α plays a critical role in anticancer drug resistance, partly through mitochondrial changes [121]. HIF1α promotes dynamin-related protein 1-dependent mitochondrial fission, facilitating the Warburg phenotype [122,123,124,125]. Divided mitochondria are observed in cells with reduced respiratory activity [126]. We previously demonstrated that HIF1α stabilization promotes reprogramming of energy metabolism and contributes to early resistance to gemcitabine treatment [127]. Under hypoxic conditions, ME1 is induced by HIF1α and participates in metabolic reprogramming [40].

Hypoxia also activates YAP via HIF1α-mediated upregulation of G protein-coupled receptor class C group 5 member A (GPRC5A) [128]. In turn, activated YAP stabilizes HIF1α and enhances its activity [129]. YAP plays a role in EMT induction and energy metabolism under hypoxic conditions [40]. Hypoxia also activates jagged canonical Notch ligand 2 (JAG2), which promotes cancer stemness and EMT through the NOTCH and AKT signaling pathways [130].

## 14. NADPH

**Figure 1:**
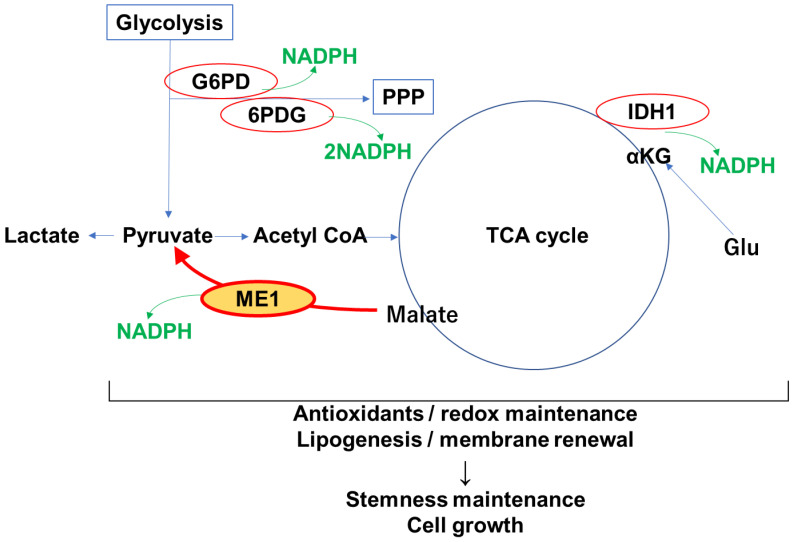
Role of ME1 in production of NADPH. NADPH is mainly produced by G6PDH and 6PDG in PPP, IDH1, and ME1, which maintain the NAPDH pool. NAPDH contributes to elimination of ROS, maintenance of redox balance, and lipogenesis, renewing membranes of cytoplasm and organelles. These roles of NADPH are essential for maintaining the stemness and proliferation of cancer cells. NADPH, nicotinamide adenine dinucleotide phosphate; ME1, malic enzyme 1; G6PD, glucose-6-phosphate dehydrogenase; 6PDG, 6-phosphogluconate dehydrogenase; IDH1, isocitrate dehydrogenase 1; αKG, α-ketoglutarate; Glu, glutamate; PPP, pentose phosphate pathway; ROS, reactive oxygen species.

NADPH is an electron donor required for reductive regeneration of antioxidant systems such as glutathione (GSH) and thioredoxin (Trx) [131]. ME1 produces NADPH during enzymatic reactions and therefore acts as a supplier of NADPH (Figure 1). In addition, G6PD and IDH1 act as NADPH suppliers (Figure 1). ME1, G6PD, and IDH1 all play important roles in metabolic pathways that are often altered in cancer cells to support their rapid growth and proliferation. In cancer cells, they are all upregulated or, like IDH1, activated by genetic mutations [132,133]. G6PD catalyzes the first reaction of the PPP and is the rate-limiting enzyme of this pathway. In the PPP, NADPH is further produced by 6-phosphogluconate dehydrogenase (6PDG). G6PD and PPP play a major role in NADPH production and contribute significantly to the NADPH pool [134]. MYC and PI3K/AKT signaling are involved in G6PD activation. ME1 and IDH1 provide alternative sources of NADPH. However, ME1 is the main supplier of NADPH in some tumors [32]. Gene expression of antioxidant pathways, including NADPH production pathways such as G6PD, 6PGD, IDH1, and ME1, is regulated by Nrf2 and is induced by increased oxidative stress [135,136]. Mutant IDH1 generates 2-hydroxyglutarate (2-HG) instead of α-ketoglutarate, increasing PPP flux [137]. Cancer cells are redundantly dependent on these pathways to meet the high demands of ROS detoxification and lipid synthesis. In CSCs, oxidative stress is a major impediment to their survival, which, together with the suppression of NADPH consumption [138], leads to increased activity of NADPH suppliers to prevent NADPH depletion [139]. A decrease in NADPH leads to fluctuations in redox balance, resulting in the accumulation of oxidative stress and cell death [106,140]. Thus, NADPH is essential for the survival of CSCs, and one of its suppliers, ME1, plays an important role in maintaining CSCs.

## 15. Carcinogenesis

Increased lipid metabolism correlates with enhanced extracellular signal-regulated kinase 2 (ERK2) activity in human patients and plays an important role in the development of CRC. Ubiquitin-specific peptidase 19 (USP19) antagonizes ring finger protein 1 (RNF1)-mediated ME1 degradation by deubiquitination, thereby promoting lipid metabolism and NADPH production, and suppressing ROS, thereby promoting colorectal carcinogenesis [141]. Wnt-mediated transcriptional activation leads to upregulation of phosphoglycerate mutase family member 5 (PGAM5) and ME1, and dephosphorylation of S336 of ME1 by PGAM5 increases ME1 K337 acetylation mediated by acetyl-CoA acetyltransferase 1 (ACAT1), leading to ME1 dimerization and activation [8]. ME1 overexpression in APC^MIN/+^ mice increases adenomas due to activation of the Wnt/β-catenin pathway and increases expression of KLF9 [34]. ME1 is also involved in hepatocarcinogenesis as one of five important prognostic genes (ME1, TP53I3, SOCS2, GADD45G, CYP7A1) associated with NASH-HCC progression [31]. In hexavalent chromium-induced lung cancer, epigenetically upregulated ME1 induces lung carcinogenesis [22]. Thus, ME1 promotes carcinogenesis by reprogramming NADPH production and metabolism.

## 16. ME1-Targeting Therapy

ME1 promotes the malignancy of various cancers by reprogramming energy metabolism, maintaining redox potential, enhancing stemness, and facilitating EMT. In particular, in low-glucose environments, cancer cells become dependent on ME1 for the supply of NADPH and pyruvate [52], ME1 has emerged as a novel molecular target for cancer therapy [142,143]. In synovial sarcoma, decreased ME1 levels promote ferroptosis by altering redox homeostasis [144].

miRNAs targeting ME1 have shown potential in suppressing cancer progression. For instance, miR30a suppresses colon carcinogenesis driven by KRAS mutations [16], while miR-885-5p suppresses gastric cancer metastasis and EMT in laryngeal SCC [26,145]. Similarly, miR-612 suppresses malignant phenotypes, including EMT, in bladder cancer [25].

Lanthanides have also been identified as ME1 inactivators, suppressing cell proliferation, motility, and EMT in OSCC cells [39]. Lanthanide administration in mouse tumor models further demonstrated suppressed tumor growth and extended survival [39].

Efforts to develop small-molecule inhibitors targeting ME1 have also shown promise. AS1134900, a novel small-molecule inhibitor, binds to a unique allosteric site outside the ME1-active site to inhibit its activity [146].

In addition, attempts have been made to discover new ME1 inhibitors. Fragment-based virtual library design and virtual screening have identified a piperazine-1-pyrrolidine-2,5-dione scaffold-based malic enzyme inhibitor that targets the NADP-binding site of malic enzyme [147].

Using a fragment-based drug discovery approach using molecular dynamic simulations and molecular docking, about 90,000 interactions were tested for the type of interaction with the NAD(P)-binding site of ME1, the binding energy, and the orientation of the compound at the site, and combination 1f was finally selected as the best inhibitor [148].

There have also been attempts to design malic enzyme inhibitors based on the X-ray crystal structure of malic enzyme, studies of pH and allosteric regulators, and the structure and dynamics of malic enzyme suggested by molecular dynamic simulations [149].

However, despite the numerous attempts mentioned above, no drugs specifically targeting ME1 have been tested in clinical trials or in late-stage clinical trials as of 2025. All cancers that overexpress ME1 have the potential to benefit from ME1-targeted therapy, making ME1 highly adaptable to cancer. Future development of ME1 inhibitors is eagerly awaited.

## 17. Conclusions

**Figure 2:**
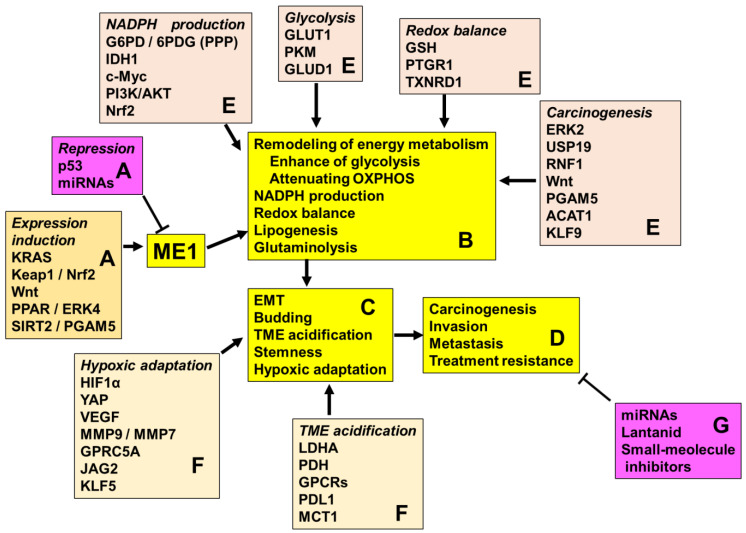
Roles of ME1 in cancer. A. Factors that affect ME1 expression. B. Biochemical changes caused by ME1 in cancer cells. C. Changes caused by ME1 in the phenotype of cancer cells. D. Changes caused by ME1 in the clinical dramatype of cancer cells. E. Factors that cooperate with the biochemical activity of ME1. F. Factors that act cooperatively on ME1-induced cancer cell phenotypes. G. Drugs targeting ME1. ME1, malic enzyme; EMT, epithelial–mesenchymal transition; NADPH, nicotinamide adenine dinucleotide phosphate; IDH1, isocitrate dehydrogenase 1; PPP, pentose phosphate pathway; G6PD, glucose-6-phosphate dehydrogenase; 6PGD, 6-phosphogluconate dehydrogenase; Keap1, Kelch-like ECH-associated protein 1; NRF2, nuclear factor erythroid 2-related factor 2; PPAR, peroxisome proliferator-activated receptor; EGR4, early growth response protein 4; SIRT2, sirtuin 2; PGAM5, phosphoglycerate mutase 5; miRNA, microRNA; PDH, pyruvate dehydrogenase; GLUT1, glucose transporter type 1; OXPHOS, oxidative phosphorylation; TME, tumor microenvironment; HIF, hypoxia-inducible factor; YAP, Yes-associated protein; PDL1, programmed death ligand 1; MCT1, monocarboxylate transporter 1; PK, pyruvate kinase; GLUD1, glutamate dehydrogenase 1; GSH, glutathione; PTGR1, prostaglandin reductase 1; TXNRD1, thioredoxin reductase 1; KLF5, Krüppel-like factor 5; VEGF, vascular endothelial growth factor; MMP, matrix metallopeptidase; GPRC5A, G protein-coupled receptor class C group 5 member A; JAG2, jagged canonical Notch ligand 2.

ME1 promotes the reprogramming of energy metabolism from OXPHOS to glycolysis and lactate fermentation, resulting in TME alterations. This shift promotes the expression of stemness-related genes, supports stemness-related metabolism, and promotes malignant phenotypes, including EMT (Figure 2). ME1 is strongly associated with poor prognosis and treatment resistance of various tumors, making it a novel therapeutic target.

## Abbreviations

ME1, malic enzyme; EMT, epithelial–mesenchymal transition; TCA cycle, tricarboxylic acid cycle; NADPH, nicotinamide adenine dinucleotide phosphate; ROS, reactive oxygen species; IDH1, isocitrate dehydrogenase 1; PPP, pentose phosphate pathway; G6PD, glucose-6-phosphate dehydrogenase; 6PGD, 6-phosphogluconate dehydrogenase; HCC, hepatocellular carcinoma; NSCLC, non-small-cell lung cancer; CRC, colorectal cancer; Keap1, Kelch-like ECH-associated protein 1; NRF2, nuclear factor erythroid 2-related factor 2; Bach-1, BTB and CNC homology 1; PPAR, peroxisome proliferator-activated receptor; EGR4, early growth response protein 4; m5C, 5-methyl cytosine; SIRT2, sirtuin 2; PGAM5, phosphoglycerate mutase 5; miRNA, microRNA; NASH, nonalcoholic steatohepatitis; SPDYC, speedy/RINGO cell cycle regulator family member C; SCC, squamous cell carcinoma; OSCC, oral SCC; ECD, E-cadherin; PDH, pyruvate dehydrogenase; GLUT1, glucose transporter type 1; OXPHOS, oxidative phosphorylation; TME, tumor microenvironment; HIF, hypoxia-inducible factor; YAP, Yes-associated protein; PD-L1, programmed death ligand 1; MCT1, monocarboxylate transporter 1; ATP, adenosine triphosphate; PK, pyruvate kinase; ACC, acetyl-coenzyme A carboxylase-α; GLUD1, glutamate dehydrogenase 1; GSH, glutathione; GSSG, glutathione SH; PTGR1, prostaglandin reductase 1; TXNRD1, thioredoxin reductase 1; IL, interleukin; JAK2, Janus kinase 2; STAT3, signal transducer and activator of transcription 3; CSC, cancer stem cell; TAZ, tafazzin; KLF5, Krüppel-like factor 5; NQO1, NAD(P)H quinone dehydrogenase 1; TRIM16, tripartite motif-containing 16; HOXA11-AS, homeobox protein Hox-A11 antisense RNA; lncRNA, long non-coding RNA; VEGF, vascular endothelial growth factor; MMP, matrix metallopeptidase; GPRC5A, G protein-coupled receptor class C group 5 member A; JAG2, jagged canonical Notch ligand 2.

## References

[1] Povey S., Wilson D.E., Harris H., Gormley I.P., Perry P., Buckton K.E. (1975). Sub-unit structure of soluble and mitochondrial malic enzyme: Demonstration of human mitochondrial enzyme in human-mouse hybrids. Ann. Hum. Genet..

[2] Frenkel R. (1975). Regulation and physiological functions of malic enzymes. Curr. Top. Cell. Regul..

[3] Merritt T.J., Kuczynski C., Sezgin E., Zhu C.T., Kumagai S., Eanes W.F. (2009). Quantifying interactions within the NADP(H) enzyme network in Drosophila melanogaster. Genetics.

[4] Rzezniczak T.Z., Merritt T.J. (2012). Interactions of NADP-reducing enzymes across varying environmental conditions: A model of biological complexity. G3 (Bethesda).

[5] Goodman R.P., Calvo S.E., Mootha V.K. (2018). Spatiotemporal compartmentalization of hepatic NADH and NADPH metabolism. J. Biol. Chem..

[6] Drake R.L., Mucenski C.G. (1985). Insulin mediates the asynchronous accumulation of hepatic albumin and malic enzyme messenger RNAs. Biochem. Biophys. Res. Commun..

[7] McHugh K.M., Drake R.L. (1989). Insulin-mediated regulation of epididymal fat pad malic enzyme. Mol. Cell Endocrinol..

[8] Zhu Y., Gu L., Lin X., Liu C., Lu B., Cui K., Zhou F., Zhao Q., Prochownik E.V., Fan C. (2020). Dynamic Regulation of ME1 Phosphorylation and Acetylation Affects Lipid Metabolism and Colorectal Tumorigenesis. Mol. Cell.

[9] Ochoa S. (1955). Malic Enzyme. Methods in Enzymology.

[10] Hsu R., Lardy H. (1967). Malic Enzyme. The Enzymes.

[11] Williamson D.H., Lund P., Krebs H.A. (1967). The redox state of free nicotinamide-adenine dinucleotide in the cytoplasm and mitochondria of rat liver. Biochem. J..

[12] Jiang P., Du W., Mancuso A., Wellen K.E., Yang X. (2013). Reciprocal regulation of p53 and malic enzymes modulates metabolism and senescence. Nature.

[13] Chou W.Y., Ho C.L., Tseng M.L., Liu S.T., Yen L.C., Huang S.M. (2008). Human Spot 14 protein is a p53-dependent transcriptional coactivator via the recruitment of thyroid receptor and Zac1. Int. J. Biochem. Cell Biol..

[14] Wen D., Liu D., Tang J., Dong L., Liu Y., Tao Z., Wan J., Gao D., Wang L., Sun H. (2015). Malic enzyme 1 induces epithelial-mesenchymal transition and indicates poor prognosis in hepatocellular carcinoma. Tumour. Biol..

[15] Chakrabarti G. (2015). Mutant KRAS associated malic enzyme 1 expression is a predictive marker for radiation therapy response in non-small cell lung cancer. Radiat. Oncol..

[16] Shen H., Xing C., Cui K., Li Y., Zhang J., Du R., Zhang X. (2017). MicroRNA-30a attenuates mutant KRAS-driven colorectal tumorigenesis via direct suppression of ME1. Cell Death Differ..

[17] Ryan E.M., Sadiku P., Coelho P., Watts E.R., Zhang A., Howden A.J.M., Sanchez-Garcia M.A., Bewley M., Cole J., McHugh B.J. (2023). NRF2 Activation Reprograms Defects in Oxidative Metabolism to Restore Macrophage Function in Chronic Obstructive Pulmonary Disease. Am. J. Respir. Crit. Care. Med..

[18] Warnatz H.J., Schmidt D., Manke T., Piccini I., Sultan M., Borodina T., Balzereit D., Wruck W., Soldatov A., Vingron M. (2011). The BTB and CNC homology 1 (BACH1) target genes are involved in the oxidative stress response and in control of the cell cycle. J. Biol. Chem..

[19] Knoblich K., Wang H.X., Sharma C., Fletcher A.L., Turley S.J., Hemler M.E. (2014). Tetraspanin TSPAN12 regulates tumor growth and metastasis and inhibits beta-catenin degradation. Cell. Mol. Life Sci..

[20] Al-Dwairi A., Pabona J.M., Simmen R.C., Simmen F.A. (2012). Cytosolic malic enzyme 1 (ME1) mediates high fat diet-induced adiposity, endocrine profile, and gastrointestinal tract proliferation-associated biomarkers in male mice. PLoS ONE.

[21] Krishnan M.L., Wang Z., Silver M., Boardman J.P., Ball G., Counsell S.J., Walley A.J., Montana G., Edwards A.D. (2016). Possible relationship between common genetic variation and white matter development in a pilot study of preterm infants. Brain Behav..

[22] Zhang R.K., Li Y., Sun F.L., Zhou Z.H., Xie Y.X., Liu W.J., Wang W., Qiu J.G., Jiang B.H., Wang L. (2024). RNA methyltransferase NSUN2-mediated m5C methylation promotes Cr(VI)-induced malignant transformation and lung cancer by accelerating metabolism reprogramming. Environ. Int..

[23] Fu G., Li S.T., Jiang Z., Mao Q., Xiong N., Li X., Hao Y., Zhang H. (2023). PGAM5 deacetylation mediated by SIRT2 facilitates lipid metabolism and liver cancer proliferation. Acta Biochim. Biophys. Sin..

[24] Zhu W., Wang H., Wei J., Sartor G.C., Bao M.M., Pierce C.T., Wahlestedt C.R., Dykxhoorn D.M., Dong C. (2018). Cocaine Exposure Increases Blood Pressure and Aortic Stiffness via the miR-30c-5p-Malic Enzyme 1-Reactive Oxygen Species Pathway. Hypertension.

[25] Liu M., Chen Y., Huang B., Mao S., Cai K., Wang L., Yao X. (2018). Tumor-suppressing effects of microRNA-612 in bladder cancer cells by targeting malic enzyme 1 expression. Int. J. Oncol..

[26] Jiang Z., Cui H., Zeng S., Li L. (2021). miR-885-5p Inhibits Invasion and Metastasis in Gastric Cancer by Targeting Malic Enzyme 1. DNA Cell Biol..

[27] Mihara Y., Akiba J., Ogasawara S., Kondo R., Fukushima H., Itadani H., Obara H., Kakuma T., Kusano H., Naito Y. (2019). Malic enzyme 1 is a potential marker of combined hepatocellular cholangiocarcinoma, subtype with stem-cell features, intermediate-cell type. Hepatol. Res..

[28] Xu K., Xia P., Liu P., Zhang X. (2022). A six lipid metabolism related gene signature for predicting the prognosis of hepatocellular carcinoma. Sci. Rep..

[29] Wang W., Zhang C., Yu Q., Zheng X., Yin C., Yan X., Liu G., Song Z. (2021). Development of a novel lipid metabolism-based risk score model in hepatocellular carcinoma patients. BMC Gastroenterol..

[30] Cao L., Liu M., Ma X., Rong P., Zhang J., Wang W. (2024). Comprehensive scRNA-seq Analysis and Identification of CD8_+T Cell Related Gene Markers for Predicting Prognosis and Drug Resistance of Hepatocellular Carcinoma. Curr. Med. Chem..

[31] Yu Q., Zhang Y., Ni J., Shen Y., Hu W. (2024). Identification and analysis of significant genes in nonalcoholic steatohepatitis-hepatocellular carcinoma transformation: Bioinformatics analysis and machine learning approach. Mol. Immunol..

[32] Lu Y.X., Ju H.Q., Liu Z.X., Chen D.L., Wang Y., Zhao Q., Wu Q.N., Zeng Z.L., Qiu H.B., Hu P.S. (2018). ME1 Regulates NADPH Homeostasis to Promote Gastric Cancer Growth and Metastasis. Cancer Res..

[33] Gdynia G., Sauer S.W., Kopitz J., Fuchs D., Duglova K., Ruppert T., Miller M., Pahl J., Cerwenka A., Enders M. (2016). The HMGB1 protein induces a metabolic type of tumour cell death by blocking aerobic respiration. Nat. Commun..

[34] Fernandes L.M., Al-Dwairi A., Simmen R.C.M., Marji M., Brown D.M., Jewell S.W., Simmen F.A. (2018). Malic Enzyme 1 (ME1) is pro-oncogenic in Apc(Min/+) mice. Sci. Rep..

[35] Liu C., Cao J., Lin S., Zhao Y., Zhu M., Tao Z., Hu X. (2020). Malic Enzyme 1 Indicates Worse Prognosis in Breast Cancer and Promotes Metastasis by Manipulating Reactive Oxygen Species. Onco. Targets. Ther..

[36] Liao R., Ren G., Liu H., Chen X., Cao Q., Wu X., Li J., Dong C. (2018). ME1 promotes basal-like breast cancer progression and associates with poor prognosis. Sci. Rep..

[37] Chen X., Peng H., Zhang Z., Yang C., Liu Y., Chen Y., Yu F., Wu S., Cao L. (2024). SPDYC serves as a prognostic biomarker related to lipid metabolism and the immune microenvironment in breast cancer. Immunol. Res..

[38] Csanadi A., Kayser C., Donauer M., Gumpp V., Aumann K., Rawluk J., Prasse A., zur Hausen A., Wiesemann S., Werner M. (2015). Prognostic Value of Malic Enzyme and ATP-Citrate Lyase in Non-Small Cell Lung Cancer of the Young and the Elderly. PLoS ONE.

[39] Nakashima C., Yamamoto K., Fujiwara-Tani R., Luo Y., Matsushima S., Fujii K., Ohmori H., Sasahira T., Sasaki T., Kitadai Y. (2018). Expression of cytosolic malic enzyme (ME1) is associated with disease progression in human oral squamous cell carcinoma. Cancer. Sci..

[40] Nakashima C., Kirita T., Yamamoto K., Mori S., Luo Y., Sasaki T., Fujii K., Ohmori H., Kawahara I., Mori T. (2020). Malic Enzyme 1 Is Associated with Tumor Budding in Oral Squamous Cell Carcinomas. Int. J. Mol. Sci..

[41] Nicolau-Neto P., de Souza-Santos P.T., Severo Ramundo M., Valverde P., Martins I., Santos I.C., Dias F., de Almeida Simão T., Ribeiro Pinto L.F. (2020). Transcriptome Analysis Identifies ALCAM Overexpression as a Prognosis Biomarker in Laryngeal Squamous Cell Carcinoma. Cancers.

[42] Zheng F.J., Ye H.B., Wu M.S., Lian Y.F., Qian C.N., Zeng Y.X. (2012). Repressing malic enzyme 1 redirects glucose metabolism, unbalances the redox state, and attenuates migratory and invasive abilities in nasopharyngeal carcinoma cell lines. Chin. J. Cancer..

[43] Huang D., Zhang C., Xiao M., Li X., Chen W., Jiang Y., Yuan Y., Zhang Y., Zou Y., Deng L. (2023). Redox metabolism maintains the leukemogenic capacity and drug resistance of AML cells. Proc. Natl. Acad. Sci. USA.

[44] Zhang C., Li W., Wu F., Lu Z., Zeng P., Luo Z., Cao Y., Wen F., Li J., Chen X. (2024). High expression of malic enzyme 1 predicts adverse prognosis in patients with cytogenetically normal acute myeloid leukaemia and promotes leukaemogenesis through the IL-6/JAK2/STAT3 pathways. Ther. Adv. Hematol..

[45] The Human Protein Atlas. https://www.proteinatlas.org/ENSG00000065833-ME1/cancer.

[46] Simmen F.A., Alhallak I., Simmen R.C.M. (2020). Malic enzyme 1 (ME1) in the biology of cancer: It is not just intermediary metabolism. J. Mol. Endocrinol..

[47] Lega I.C., Lipscombe L.L. (2020). Review: Diabetes, Obesity, and Cancer-Pathophysiology and Clinical Implications. Endocr. Rev..

[48] Fan Y., He J., Shi L., Zhang M., Chen Y., Xu L., Han N., Jiang Y. (2024). Identification of potential key lipid metabolism-related genes involved in tubular injury in diabetic kidney disease by bioinformatics analysis. Acta Diabetol..

[49] Zhang H., Wu J., Li Y., Jin G., Tian Y., Kang S. (2022). Identification of Key Differentially Methylated/Expressed Genes and Pathways for Ovarian Endometriosis by Bioinformatics Analysis. Reprod. Sci..

[50] Meng L.B., Hu G.F., Lv T., Lv C., Liu L., Zhang P. (2024). Higher expression of TSR2 aggravating hypertension via the PPAR signaling pathway. Aging.

[51] Yao B., Zhang Y., Wu Q., Yao H., Peng L., Jiang Z., Yang L., Yuan L. (2024). Comprehensive assessment of cellular senescence in intestinal immunity and biologic therapy response in ulcerative colitis. Sci. Rep..

[52] Murai S., Ando A., Ebara S., Hirayama M., Satomi Y., Hara T. (2017). Inhibition of malic enzyme 1 disrupts cellular metabolism and leads to vulnerability in cancer cells in glucose-restricted conditions. Oncogenesis.

[53] Shi Y., Zhou S., Wang P., Guo Y., Xie B., Ding S. (2019). Malic enzyme 1 (ME1) is a potential oncogene in gastric cancer cells and is associated with poor survival of gastric cancer patients. Onco. Targets. Ther..

[54] Kourtidis A., Jain R., Carkner R.D., Eifert C., Brosnan M.J., Conklin D.S. (2010). An RNA interference screen identifies metabolic regulators NR1D1 and PBP as novel survival factors for breast cancer cells with the ERBB2 signature. Cancer Res..

[55] Yao P., Sun H., Xu C., Chen T., Zou B., Jiang P., Du W. (2017). Evidence for a direct cross-talk between malic enzyme and the pentose phosphate pathway via structural interactions. J. Biol. Chem..

[56] Woo S.H., Yang L.P., Chuang H.C., Fitzgerald A., Lee H.Y., Pickering C., Myers J.N., Skinner H.D. (2016). Down-regulation of malic enzyme 1 and 2: Sensitizing head and neck squamous cell carcinoma cells to therapy-induced senescence. Head. Neck..

[57] Yang J., Antin P., Berx G., Blanpain C., Brabletz T., Bronner M., Campbell K., Cano A., Casanova J., Christofori G. (2020). Guidelines and definitions for research on epithelial-mesenchymal transition. Nat. Rev. Mol. Cell Biol..

[58] Liu J.F., Mao L., Bu L.L., Ma S.R., Huang C.F., Zhang W.F., Sun Z.J. (2015). C4.4A as a biomarker of head and neck squamous cell carcinoma and correlated with epithelial mesenchymal transition. Am. J. Cancer Res..

[59] Justus C.R., Dong L., Yang L.V. (2013). Acidic tumor microenvironment and pH-sensing G protein-coupled receptors. Front. Physiol..

[60] Prall F. (2007). Tumour budding in colorectal carcinoma. Histopathology.

[61] Kanazawa H., Mitomi H., Nishiyama Y., Kishimoto I., Fukui N., Nakamura T., Watanabe M. (2008). Tumour budding at invasive margins and outcome in colorectal cancer. Color. Dis..

[62] Mitrovic B., Schaeffer D.F., Riddell R.H., Kirsch R. (2012). Tumor budding in colorectal carcinoma: Time to take notice. Mod. Pathol..

[63] Almangush A., Pirinen M., Heikkinen I., Mäkitie A.A., Salo T., Leivo I. (2018). Tumour budding in oral squamous cell carcinoma: A meta-analysis. Br. J. Cancer.

[64] Yamakawa N., Kirita T., Umeda M., Yanamoto S., Ota Y., Otsuru M., Okura M., Kurita H., Yamada S.I., Hasegawa T. (2019). Tumor budding and adjacent tissue at the invasive front correlate with delayed neck metastasis in clinical early-stage tongue squamous cell carcinoma. J. Surg. Oncol..

[65] Elseragy A., Salo T., Coletta R.D., Kowalski L.P., Haglund C., Nieminen P., Mäkitie A.A., Leivo I., Almangush A. (2019). A Proposal to Revise the Histopathologic Grading System of Early Oral Tongue Cancer Incorporating Tumor Budding. Am. J. Surg. Pathol..

[66] Li H., Xu F., Li S., Zhong A., Meng X., Lai M. (2016). The tumor microenvironment: An irreplaceable element of tumor budding and epithelial-mesenchymal transition-mediated cancer metastasis. Cell Adh. Migr..

[67] Grigore A.D., Jolly M.K., Jia D., Farach-Carson M.C., Levine H. (2016). Tumor Budding: The Name is EMT. Partial EMT. J. Clin. Med..

[68] Mezheyeuski A., Nerovnya A., Bich T., Tur G., Ostman A., Portyanko A. (2015). Inter- and intra-tumoral relationships between vasculature characteristics, GLUT1 and budding in colorectal carcinoma. Histol. Histopathol..

[69] Dong C., Yuan T., Wu Y., Wang Y., Fan T.W., Miriyala S., Lin Y., Yao J., Shi J., Kang T. (2013). Loss of FBP1 by Snail-mediated repression provides metabolic advantages in basal-like breast cancer. Cancer Cell.

[70] Georges R., Bergmann F., Hamdi H., Zepp M., Eyol E., Hielscher T., Berger M.R., Adwan H. (2012). Sequential biphasic changes in claudin1 and claudin4 expression are correlated to colorectal cancer progression and liver metastasis. J. Cell. Mol. Med..

[71] McDonald P.C., Chafe S.C., Dedhar S. (2016). Overcoming Hypoxia-Mediated Tumor Progression: Combinatorial Approaches Targeting pH Regulation, Angiogenesis and Immune Dysfunction. Front. Cell Dev. Biol..

[72] Vaitheesvaran B., Xu J., Yee J., Q-Y L., Go V.L., Xiao G.G., Lee W.N. (2015). The Warburg effect: A balance of flux analysis. Metabolomics.

[73] Icard P., Kafara P., Steyaert J.M., Schwartz L., Lincet H. (2014). The metabolic cooperation between cells in solid cancer tumors. Biochim. Biophys. Acta.

[74] Warburg O. (1956). On respiratory impairment in cancer cells. Science.

[75] Otto-Warburg O. (1956). On the origin of cancer cells. Science.

[76] Beisan-Lu J., Tan M., Cai Q. (2015). The Warburg effect in tumor progression: Mitochondrial oxidative metabolism as an anti-metastasis mechanism. Cancer Lett..

[77] Barclay C.J. (2017). Energy demand and supply in human skeletal muscle. J. Muscle Res. Cell Motil..

[78] Rose I.A. (1998). How fumarase recycles after the malate --> fumarate reaction. Insights into the reaction mechanism. Biochemistry.

[79] Wehrle J.P., Ng C.E., McGovern K.A., Aiken N.R., Shungu D.C., Chance E.M., Glickson J.D. (2000). Metabolism of alternative substrates and the bioenergetic status of EMT6 tumor cell spheroids. NMR Biomed..

[80] Luo Y., Yoneda J., Ohmori H., Sasaki T., Shimbo K., Eto S., Kato Y., Miyano H., Kobayashi T., Sasahira T. (2014). Cancer usurps skeletal muscle as an energy repository. Cancer Res..

[81] Son J., Lyssiotis C.A., Ying H., Wang X., Hua S., Ligorio M., Perera R.M., Ferrone C.R., Mullarky E., Shyh-Chang N. (2013). Glutamine supports pancreatic cancer growth through a KRAS-regulated metabolic pathway. Nature.

[82] Reitzer L.J., Wice B.M., Kennell D. (1979). Evidence that glutamine, not sugar, is the major energy source for cultured HeLa cells. J. Biol. Chem..

[83] Sarfraz I., Rasul A., Hussain G., Shah M.A., Zahoor A.F., Asrar M., Selamoglu Z., Ji X.Y., Adem Ş., Sarker S.D. (2020). 6-Phosphogluconate dehydrogenase fuels multiple aspects of cancer cells: From cancer initiation to metastasis and chemoresistance. Biofactors.

[84] Wallace D.C. (2005). Mitochondria and cancer: Warburg addressed. Cold. Spring. Harb. Symp. Quant. Biol..

[85] Fujiwara-Tani R., Sasaki T., Takagi T., Mori S., Kishi S., Nishiguchi Y., Ohmori H., Fujii K., Kuniyasu H. (2022). Gemcitabine Resistance in Pancreatic Ductal Carcinoma Cell Lines Stems from Reprogramming of Energy Metabolism. Int. J. Mol. Sci..

[86] Lee D., Zhang M.S., Tsang F.H., Bao M.H., Xu I.M., Lai R.K., Chiu D.K., Tse A.P., Law C.T., Chan C.Y. (2021). Adaptive and Constitutive Activations of Malic Enzymes Confer Liver Cancer Multilayered Protection Against Reactive Oxygen Species. Hepatology.

[87] Rocha C.M., Barros A.S., Goodfellow B.J., Carreira I.M., Gomes A., Sousa V., Bernardo J., Carvalho L., Gil A.M., Duarte I.F. (2015). NMR metabolomics of human lung tumours reveals distinct metabolic signatures for adenocarcinoma and squamous cell carcinoma. Carcinogenesis.

[88] Yang C.S., Thomenius M.J., Gan E.C., Tang W., Freel C.D., Merritt T.J., Nutt L.K., Kornbluth S. (2010). Metabolic regulation of Drosophila apoptosis through inhibitory phosphorylation of Dronc. EMBO J..

[89] Ran M., Zhou Y., Guo Y., Huang D., Zhang S.L., Tam K.Y. (2023). Cytosolic malic enzyme and glucose-6-phosphate dehydrogenase modulate redox balance in NSCLC with acquired drug resistance. FEBS J..

[90] Pastushenko I., Blanpain C. (2019). EMT Transition States during Tumor Progression and Metastasis. Trends Cell. Biol..

[91] Brabletz S., Schuhwerk H., Brabletz T., Stemmler M.P. (2021). Dynamic EMT: A multi-tool for tumor progression. EMBO J..

[92] Loh J.J., Ma S. (2024). Hallmarks of cancer stemness. Cell Stem Cell.

[93] Nallasamy P., Nimmakayala R.K., Parte S., Are A.C., Batra S.K., Ponnusamy M.P. (2022). Tumor microenvironment enriches the stemness features: The architectural event of therapy resistance and metastasis. Mol. Cancer.

[94] Shao C., Lu W., Du Y., Yan W., Bao Q., Tian Y., Wang G., Ye H., Hao H. (2020). Cytosolic ME1 integrated with mitochondrial IDH2 supports tumor growth and metastasis. Redox. Biol..

[95] Weiss F., Lauffenburger D., Friedl P. (2022). Towards targeting of shared mechanisms of cancer metastasis and therapy resistance. Nat. Rev. Cancer.

[96] Walcher L., Kistenmacher A.K., Suo H., Kitte R., Dluczek S., Strauß A., Blaudszun A.R., Yevsa T., Fricke S., Kossatz-Boehlert U. (2020). Cancer Stem Cells-Origins and Biomarkers: Perspectives for Targeted Personalized Therapies. Front. Immunol..

[97] Jing N., Gao W.Q., Fang Y.X. (2021). Regulation of Formation, Stemness and Therapeutic Resistance of Cancer Stem Cells. Front. Cell Dev. Biol..

[98] Stockard B., Bhise N., Shin M., Guingab-Cagmat J., Garrett T.J., Pounds S., Lamba J.K. (2021). Cellular Metabolomics Profiles Associated with Drug Chemosensitivity in AML. Front. Oncol..

[99] Cui Y., Nadiminty N., Liu C., Lou W., Schwartz C.T., Gao A.C. (2014). Upregulation of glucose metabolism by NF-κB2/p52 mediates enzalutamide resistance in castration-resistant prostate cancer cells. Endocr. Relat. Cancer.

[100] Wong C.C., Xu J., Bian X., Wu J.L., Kang W., Qian Y., Li W., Chen H., Gou H., Liu D. (2020). In Colorectal Cancer Cells with Mutant KRAS, SLC25A22-Mediated Glutaminolysis Reduces DNA Demethylation to Increase WNT Signaling, Stemness, and Drug Resistance. Gastroenterology.

[101] Deshmukh A., Deshpande K., Arfuso F., Newsholme P., Dharmarajan A. (2016). Cancer stem cell metabolism: A potential target for cancer therapy. Mol. Cancer.

[102] Kao T.W., Chuang Y.C., Lee H.L., Kuo C.C., Shen Y.A. (2022). Therapeutic Targeting of Glutaminolysis as a Novel Strategy to Combat Cancer Stem Cells. Int. J. Mol. Sci..

[103] Koo J.H., Guan K.L. (2018). Interplay between YAP/TAZ and Metabolism. Cell Metab..

[104] Bhattacharya D., Azambuja A.P., Simoes-Costa M. (2020). Metabolic Reprogramming Promotes Neural Crest Migration via Yap/Tead Signaling. Dev. Cell.

[105] Tang Y., Feinberg T., Keller E.T., Li X.Y., Weiss S.J. (2016). Snail/Slug binding interactions with YAP/TAZ control skeletal stem cell self-renewal and differentiation. Nat. Cell Biol..

[106] Chen H., Hou S., Zhang H., Zhou B., Xi H., Li X., Lufeng Z., Guo Q. (2024). MiR-375 impairs breast cancer cell stemness by targeting the KLF5/G6PD signaling axis. Environ. Toxicol..

[107] Kahroba H., Shirmohamadi M., Hejazi M.S., Samadi N. (2019). The Role of Nrf2 signaling in cancer stem cells: From stemness and self-renewal to tumorigenesis and chemoresistance. Life Sci..

[108] Nakashima C., Fujiwara-Tani R., Mori S., Kishi S., Ohmori H., Fujii K., Mori T., Miyagawa Y., Yamamoto K., Kirita T. (2022). An Axis between the Long Non-Coding RNA HOXA11-AS and NQOs Enhances Metastatic Ability in Oral Squamous Cell Carcinoma. Int. J. Mol. Sci..

[109] Chen D., Sun Q., Zhang L., Zhou X., Cheng X., Zhou D., Ye F., Lin J., Wang W. (2017). The lncRNA HOXA11-AS functions as a competing endogenous RNA to regulate PADI2 expression by sponging miR-125a-5p in liver metastasis of colorectal cancer. Oncotarget.

[110] Wang S., Zhang S., He Y., Huang X., Hui Y., Tang Y. (2019). HOXA11-AS regulates JAK-STAT pathway by miR-15a-3p/STAT3 axis to promote the growth and metastasis in liver cancer. J. Cell. Biochem..

[111] Hockel M., Schlenger K., Aral B., Mitze M., Schaffer U., Vaupel P. (1996). Association between tumor hypoxia and malignant progression in advanced cancer of the uterine cervix. Cancer. Res..

[112] Simonetti O., Lucarini G., Rubini C., Goteri G., Zizzi A., Staibano S., Campanati A., Ganzetti G., Di Primio R., Offidani A. (2013). Microvessel density and VEGF, HIF-1α expression in primary oral melanoma: Correlation with prognosis. Oral. Dis..

[113] Datcher-Li Y.Y., Zheng Y.L. (2017). Hypoxia promotes invasion of retinoblastoma cells in vitro by upregulating HIF-1α/MMP9 signaling pathway. Eur. Rev. Med. Pharmacol. Sci..

[114] Liu H.L., Liu D., Ding G.R., Liao P.F., Zhang J.W. (2015). Hypoxia-inducible factor-1α and Wnt/β-catenin signaling pathways promote the invasion of hypoxic gastric cancer cells. Mol. Med. Rep..

[115] Wu Z., Zuo M., Zeng L., Cui K., Liu B., Yan C., Chen L., Dong J., Shangguan F., Hu W. (2021). OMA1 reprograms metabolism under hypoxia to promote colorectal cancer development. EMBO Rep..

[116] Elvidge G.P., Glenny L., Appelhoff R.J., Ratcliffe P.J., Ragoussis J., Gleadle J.M. (2006). Concordant regulation of gene expression by hypoxia and 2-oxoglutarate-dependent dioxygenase inhibition: The role of HIF-1alpha, HIF-2alpha, and other pathways. J. Biol. Chem..

[117] Al Tameemi W., Dale T.P., Al-Jumaily R.M.K., Forsyth N.R. (2019). Hypoxia-Modified Cancer Cell Metabolism. Front. Cell Dev. Biol..

[118] Ishihara N., Nomura M., Jofuku A., Kato H., Suzuki S.O., Masuda K., Otera H., Nakanishi Y., Nonaka I., Goto Y. (2009). Mitochondrial fission factor Drp1 is essential for embryonic development and synapse formation in mice. Nat. Cell Biol..

[119] Wakabayashi J., Zhang Z., Wakabayashi N., Tamura Y., Fukaya M., Kensler T.W., Iijima M., Sesaki H. (2009). The dynamin-related GTPase Drp1 is required for embryonic and brain development in mice. J. Cell Biol..

[120] Plecitá-Hlavatá L., Engstová H., Alán L., Špaček T., Dlasková A., Smolková K., Špačková J., Tauber J., Strádalová V., Malínský J. (2016). Hypoxic HepG2 cell adaptation decreases ATP synthase dimers and ATP production in inflated cristae by mitofilin down-regulation concomitant to MICOS clustering. FASEB J..

[121] Chiche J., Rouleau M., Gounon P., Brahimi-Horn M.C., Pouysségur J., Mazure N.M. (2010). Hypoxic enlarged mitochondria protect cancer cells from apoptotic stimuli. J. Cell. Physiol..

[122] Marsboom G., Toth P.T., Ryan J.J., Hong Z., Wu X., Fang Y.H., Thenappan T., Piao L., Zhang H.J., Pogoriler J. (2012). Dynamin-related protein 1-mediated mitochondrial mitotic fission permits hyperproliferation of vascular smooth muscle cells and offers a novel therapeutic target in pulmonary hypertension. Circ. Res..

[123] Elstrom R.L., Bauer D.E., Buzzai M., Karnauskas R., Harris M.H., Plas D.R., Zhuang H., Cinalli R.M., Alavi A., Rudin C.M. (2004). Akt stimulates aerobic glycolysis in cancer cells. Cancer Res..

[124] Mauro C., Leow S.C., Anso E., Rocha S., Thotakura A.K., Tornatore L., Moretti M., De Smaele E., Beg A.A., Tergaonkar V. (2011). NF-κB controls energy homeostasis and metabolic adaptation by upregulating mitochondrial respiration. Nat. Cell Biol..

[125] Papandreou I., Cairns R.A., Fontana L., Lim A.L., Denko N.C. (2006). HIF-1 mediates adaptation to hypoxia by actively downregulating mitochondrial oxygen consumption. Cell Metab..

[126] Westermann B. (2012). Bioenergetic role of mitochondrial fusion and fission. Biochim. Biophys. Acta.

[127] Takagi T., Fujiwara-Tani R., Mori S., Kishi S., Nishiguchi Y., Sasaki T., Ogata R., Ikemoto A., Sasaki R., Ohmori H. (2023). Lauric Acid Overcomes Hypoxia-Induced Gemcitabine Chemoresistance in Pancreatic Ductal Adenocarcinoma. Int. J. Mol. Sci..

[128] Greenhough A., Bagley C., Heesom K.J., Gurevich D.B., Gay D., Bond M., Collard T.J., Paraskeva C., Martin P., Sansom O.J. (2018). Cancer cell adaptation to hypoxia involves a HIF-GPRC5A-YAP axis. EMBO Mol. Med..

[129] Zhang X., Li Y., Ma Y., Yang L., Wang T., Meng X., Zong Z., Sun X., Hua X., Li H. (2018). Yes-associated protein (YAP) binds to HIF-1α and sustains HIF-1α protein stability to promote hepatocellular carcinoma cell glycolysis under hypoxic stress. J. Exp. Clin. Cancer Res..

[130] Xing F., Okuda H., Watabe M., Kobayashi A., Pai S.K., Liu W., Pandey P.R., Fukuda K., Hirota S., Sugai T. (2011). Hypoxia-induced Jagged2 promotes breast cancer metastasis and self-renewal of cancer stem-like cells. Oncogene.

[131] Xiao W., Loscalzo J. (2020). Metabolic Responses to Reductive Stress. Antioxid. Redox. Signal..

[132] Song J., Sun H., Zhang S., Shan C. (2022). The Multiple Roles of Glucose-6-Phosphate Dehydrogenase in Tumorigenesis and Cancer Chemoresistance. Life.

[133] Dang L., White D.W., Gross S., Bennett B.D., Bittinger M.A., Driggers E.M., Fantin V.R., Jang H.G., Jin S., Keenan M.C. (2009). Cancer-associated IDH1 mutations produce 2-hydroxyglutarate. Nature.

[134] Bhanot H., Weisberg E.L., Reddy M.M., Nonami A., Neuberg D., Stone R.M., Podar K., Salgia R., Griffin J.D., Sattler M. (2017). Acute myeloid leukemia cells require 6-phosphogluconate dehydrogenase for cell growth and NADPH-dependent metabolic reprogramming. Oncotarget.

[135] Qaisiya M., Coda Zabetta C.D., Bellarosa C., Tiribelli C. (2014). Bilirubin mediated oxidative stress involves antioxidant response activation via Nrf2 pathway. Cell Signal..

[136] He X., Zhou Y., Chen W., Zhao X., Duan L., Zhou H., Li M., Yu Y., Zhao J., Guo Y. (2023). Repurposed pizotifen malate targeting NRF2 exhibits anti-tumor activity through inducing ferroptosis in esophageal squamous cell carcinoma. Oncogene.

[137] Gelman S.J., Naser F., Mahieu N.G., McKenzie L.D., Dunn G.P., Chheda M.G., Patti G.J. (2018). Consumption of NADPH for 2-HG Synthesis Increases Pentose Phosphate Pathway Flux and Sensitizes Cells to Oxidative Stress. Cell Rep..

[138] Wei Q., Qian Y., Yu J., Wong C.C. (2020). Metabolic rewiring in the promotion of cancer metastasis: Mechanisms and therapeutic implications. Oncogene.

[139] Smolková K., Plecitá-Hlavatá L., Bellance N., Benard G., Rossignol R., Ježek P. (2011). Waves of gene regulation suppress and then restore oxidative phosphorylation in cancer cells. Int. J. Biochem. Cell Biol..

[140] Wang J., Chen J., Fan K., Wang M., Gao M., Ren Y., Wu S., He Q., Tu K., Xu Q. (2024). Inhibition of Endoplasmic Reticulum Stress Cooperates with SLC7A11 to Promote Disulfidptosis and Suppress Tumor Growth upon Glucose Limitation. Adv. Sci..

[141] Zhu Y., Gu L., Lin X., Zhou X., Lu B., Liu C., Lei C., Zhou F., Zhao Q., Prochownik E.V. (2021). USP19 exacerbates lipogenesis and colorectal carcinogenesis by stabilizing ME1. Cell Rep..

[142] Wang H., Cui W., Yue S., Zhu X., Li X., He L., Zhang M., Yang Y., Wei M., Wu H. (2024). Malic enzymes in cancer: Regulatory mechanisms, functions, and therapeutic implications. Redox. Biol..

[143] Lau A.N., Li Z., Danai L.V., Westermark A.M., Darnell A.M., Ferreira R., Gocheva V., Sivanand S., Lien E.C., Sapp K.M. (2020). Dissecting cell-type-specific metabolism in pancreatic ductal adenocarcinoma. Elife.

[144] Brashears C.B., Prudner B.C., Rathore R., Caldwell K.E., Dehner C.A., Buchanan J.L., Lange S.E.S., Poulin N., Sehn J.K., Roszik J. (2022). Malic Enzyme 1 Absence in Synovial Sarcoma Shifts Antioxidant System Dependence and Increases Sensitivity to Ferroptosis Induction with ACXT-3102. Clin. Cancer Res..

[145] Xu Q., Yu B., Chen W., Li W., Sun Y., Fang Y. (2022). CircSERPINA3 promoted cell proliferation, migration, and invasion of laryngeal squamous cell carcinoma by targeting miR-885-5p. Cell Biol. Int..

[146] Yoshida T., Kawabe T., Cantley L.C., Lyssiotis C.A. (2022). Discovery and Characterization of a Novel Allosteric Small-Molecule Inhibitor of NADP(+)-Dependent Malic Enzyme 1. Biochemistry.

[147] Zhang Y.J., Wang Z., Sprous D., Nabioullin R. (2006). In silico design and synthesis of piperazine-1-pyrrolidine-2,5-dione scaffold-based novel malic enzyme inhibitors. Bioorg. Med. Chem. Lett..

[148] Ramezani F. (2018). New Malic Enzyme 1 Inhibitor Design Using Fragmental-Based Virtual Screening and Molecular Dynamic Simulation. SM J. Pharmacol. Ther..

[149] Burley K.H., Cuthbert B.J., Basu P., Newcombe J., Irimpan E.M., Quechol R., Foik I.P., Mobley D.L., Beste D.J.V., Goulding C.W. (2021). Structural and Molecular Dynamics of Mycobacterium tuberculosis Malic Enzyme, a Potential Anti-TB Drug Target. ACS Infect. Dis..

